# Early Life Stress and Pediatric Posttraumatic Stress Disorder

**DOI:** 10.3390/brainsci10030169

**Published:** 2020-03-14

**Authors:** Panagiota Pervanidou, Gerasimos Makris, George Chrousos, Agorastos Agorastos

**Affiliations:** 1 Unit of Developmental and Behavioral Pediatrics, First Department of Pediatrics, School of Medicine, National and Kapodistrian University of Athens, “Aghia Sophia” Children’s Hospital, 11527 Athens, Greece; makrisgi@med.uoa.gr (G.M.); chrousge@med.uoa.gr (G.C.); 2 Department of Psychiatry, Division of Neurosciences, School of Medicine, Faculty of Health Sciences, Aristotle University of Thessaloniki, 54124 Thessaloniki, Greece; aagorast@auth.gr

**Keywords:** children, adolescents, early life stress, posttraumatic stress disorder, cortisol, catecholamines, stress, HPA axis, autonomic nervous system

## Abstract

Traumatic stress exposure during critical periods of development may have essential and long-lasting effects on the physical and mental health of individuals. Two thirds of youth are exposed to potentially traumatic experiences by the age of 17, and approximately 5% of adolescents meet lifetime criteria for posttraumatic stress disorder (PTSD). The role of the stress system is the maintenance of homeostasis in the presence of real/perceived and acute/chronic stressors. Early-life stress (ELS) has an impact on neuronal brain networks involved in stress reactions, and could exert a programming effect on glucocorticoid signaling. Studies on pediatric PTSD reveal diverse neuroendocrine responses to adverse events and related long-term neuroendocrine and epigenetic alterations. Neuroendocrine, neuroimaging, and genetic studies in children with PTSD and ELS experiences are crucial in understanding risk and resilience factors, and also the natural history of PTSD.

## 1. Introduction

The term early-life stress (ELS) has been used to describe a broad spectrum of adverse and stressful events, including childhood trauma occurring during neonatal life, early and late childhood, and adolescence. These adverse experiences include maltreatment, neglect, separation, parental loss, starvation, extreme poverty, bullying, domestic/community/school violence etc. ELS also includes adverse exposures during fetal life including maternal undernutrition, day-to-day hassles, pregnancy-specific stressors, moderate increases in maternal anxiety, and exposure of the mother to extreme adverse experiences such as physical/sexual abuse, acts of terrorism, war or natural disasters [1]. On the other hand, the term childhood trauma (CT) has been used to describe a more specific form of ELS. According to the fifth edition of the Diagnostic and Statistical Manual (DSM) of Mental Disorders (American Psychiatric Association (APA), 2013), regarding children and adolescents, trauma is defined as “exposure to actual or threatened death, serious injury, or sexual violence in one or more of the following ways: (i) Directly experiencing the traumatic event(s); (ii) witnessing, in person, the event(s) as it occurred to others; (iii) learning that the traumatic event(s) occurred to a close family member or close friend (for adolescents and children older than 6 years), or learning that the traumatic event(s) occurred to a parent or caregiving figure (for children 6 years and younger). Therefore, the term ELS describes a broader spectrum of adverse and stressful events compared to the term “trauma”, which can occur from fetal life through adolescence and which are not necessarily traumatic in the sense of the definition provided in the DSM-5.

The neuropsychobiological impact of ELS constitutes a major developmental risk factor for increased physical and mental morbidity in later life [2,3]. There is abundant evidence from both retrospective and prospective studies indicating the negative impact of ELS on adult mental [4,5,6,7,8,9,10] and physical health [3,11,12,13,14]. Concerning the mental health consequences of ELS, a higher risk of depression [6,7,8], posttraumatic stress disorder (PTSD) [4,9], schizophrenia [10], suicide attempts [5], and risky behavior patterns (e.g., tobacco and alcohol consumption) [14] in later life have been closely associated with ELS experiences. However, it has been suggested that the factors that increase the risk of trauma exposure later in life may be different than those which sensitize individuals to the adverse effects of later stressors [4].

Exposure to traumatic events is rather common in childhood and adolescence, and PTSD will occur in a significant portion of those exposed to adverse events [15]. Estimates of the incidence of PTSD in children vary by sample, different assessment methodologies, and different types of traumatic event [16]. Roughly two thirds (61.8%) of youth are exposed to trauma by 17 years of age, and approximately 5% of children and adolescents below 18 years of age meet lifetime criteria for PTSD [17,18]. PTSD prevalence below the age of 10 years has not been well studied [19].

Research and clinical practice are facilitated by a common definition of posttraumatic stress disorder (PTSD), which is very common in the general population and can occur at any age. In general terms, PTSD describes a cluster of symptoms that develop after direct or indirect exposure to traumatic life events involving actual or threatened death, serious injury, or sexual violence. PTSD was classified as an anxiety disorder in the fourth edition of the DSM (APA, 1994). More recently, it was reconceptualized in order to include a broader spectrum of negative responses to traumatic experiences. The DSM-5 relocated the disorder to a new chapter of trauma- and stressor-related disorders. A subtype was also created for children 6 years and younger, lowering the diagnostic thresholds for children. The PTSD symptoms are organized into four categories for adults, adolescents, and children older than 6 years, with one or more symptoms required from each group: (i) intrusion, (ii) avoidance, (iii) negative alterations in cognitions and mood, and (iv) alterations in arousal and reactivity. By definition, symptoms must persist for at least 1 month and cause clinically significant distress or impairment in several areas of functioning. Until recently, the diagnostic criteria of PTSD were not developmentally appropriate for diagnosing PTSD in very young children. However, the DSM-5 provides an extensive description of developmental differences in symptom expression and developmentally modified criteria for children aged 6 years old and younger. This set of developmentally sensitive criteria includes three symptom groups: (i) intrusion, (ii) persistent avoidance/negative alterations in cognition and mood, and (iii) alterations in arousal and reactivity. 

The effects of adverse and stressful events early in life may be severe and long-lasting. They are also associated with several clinical conditions including attention deficit hyperactivity disorder (ADHD), PTSD, bipolar disorder, panic disorder, substance abuse, generalized anxiety disorder (GAD), and major depressive disorder (MDD), as well as psychotic symptoms [20,21]. Depression appears to be the most common comorbid diagnosis of PTSD, with a range of 13% to 75%, whereas GAD was the comorbid diagnosis in 27.6% of the PTSD population in one study conducted in adolescents [22]. 

## 2. Stress System Components

The role of the stress system is the maintenance of homeostasis in the presence of real or perceived and acute or chronic stressors. In general, activation of the stress system leads to adequate behavioral and physical adaptive changes that improve the organism’s ability to survive. The human stress system involves central and peripheral components which drive adaptive responses via a plethora of neurotransmitters, steroid hormones, and neuropeptides. The central CNS effectors of the stress system include: (a) the corticotropin-releasing hormone (CRH) secreted by the CRH neurons. The highest concentration of CRH neurons is present in the paraventricular nucleus (PVN) of the hypothalamus. CRH is also present in limbic structures and other brain regions related to the stress response such as the bed nucleus of the stria terminalis, the central nucleus of the amygdala, the locus coeruleus, the cerebral cortex, the cerebellum, and the dorsal root neurons of the spinal cord. Moreover, CRH has been found in chromaffin cells of the adrenal medulla and sympathetic ganglia of the autonomic nervous system (ANS), and finally in peripheral organs such as immune cells, skin, and gastrointestinal tract [23]; (b) arginine–vasopressin (AVP), produced by neurons of the hypothalamic PVN; (c) the proopiomelanocortin-derived peptides α-melanocyte-stimulating hormone (MSH) and β-endorphin produced in the hypothalamic arcuate nucleus; and (d) norepinephrine produced in the A1/A2 centers of the brainstem’s LC and in the noradrenergic (NE) cell groups residing in portions of the medulla oblongata (LC/NE system). The principal peripheral effectors of the stress system are (a) the hypothalamic–pituitary–adrenal axis (HPA)-regulated glucocorticoids, and (b) the catecholamines norepinephrine and epinephrine, which are regulated by the ANS, comprising (i) the sympathetic nervous system (SNS) and the sympathoadrenomedullary (SAM) system, and (ii) the parasympathetic system (PNS). In addition, a variety of molecules, e.g., neurotransmitters, steroid hormones, and neuropeptides interact with the classic neuroendocrine hormones to maintain homeostasis [2,24].

The ANS and the hypothalamic–pituitary–adrenal (HPA) axis are regarded as the key systems involved in the stress response. The immediate response to a stressor is mediated by the instantaneous activation of the ANS and the increase of catecholamines in the periphery [25]. In response to stress, CRH is released into the hypothalamo-pituitary portal vessels and induces the synthesis and release of adrenocorticotropin hormone (ACTH) into the systemic circulation from the anterior pituitary. It has also been demonstrated that CRH itself elicits behaviors that are reminiscent of a typical stress response, and that it directly modulates the rapid response of the ANS [26]. The principal target for ACTH is the adrenal cortex, where it stimulates the release of glucocorticoids (GCs), particularly cortisol in humans [27,28]. GCs participate in a multiplicity of functions which are essential for the maintenance, duration, and downregulation of the stress response mainly through GC binding to glucocorticoid receptors (GRs) in the hippocampus [26].

The HPA axis and the ANS are complementary and closely interconnected components of an internal neural regulation system (central autonomic network, CAN) [2]. The dysregulation of the CAN may affect autonomic core centers, comprised of a set of neural structures including the prefrontal cortex (PFC), the amygdala, the hypothalamus and the brain stem, thereby altering peripheral ANS activity and overall stress responsiveness. Moreover, it has been shown that the HPA axis regulation depends in part on the ANS, especially on vagal influences [29].

Excessive and prolonged activation of the stress system might lead to increased and prolonged production of CRH, cortisol, and catecholamines, which could explain many of the long-term psychopathological and physical complications of chronic stress, such as depression, obsessive-compulsive disorder, panic anxiety, anorexia nervosa, and chronic active alcoholism, amongst others [28,30]. However, hypo-activation of the HPA axis has been reported in several repeated or chronic stress situations including PTSD, chronic fatigue syndrome, fibromyalgia, the postpartum period, and nicotine withdrawal syndrome [28]. The direction of HPA axis adaptation to chronic stress may depend on the individual’s genetic background, their previous stress history, and the nature, chronicity, severity, and predictability of the stressor. Additionally, age and developmental stage have been suggested as key determinants not only of the clinical manifestations of chronic stress, but also of the biological patterns of disease [19].

## 3. The Effects of ELS across the Lifespan 

The effects of ELS depend on three main factors: (i) the developmental time window affected [31], (ii) the sex of the individual [32], and (iii) the developmental stage at which effects are assessed [33]. Early-life stress during fetal life, childhood, and adolescence has been recognized as a key mediator of developmental programming [1]. The programming effects of ELS on the developing brain mainly concern brain networks involved in stress reactions, such as the prefrontal cortex, the hippocampus, and the amygdala, and may lead to sustained hyper- or hypo-activation of the stress system to ensuing stressors [34]. Animal studies have shown that chronic stress may affect brain development through mechanisms of accelerated loss of neurons, delays in myelination, or abnormalities in neural synaptic pruning [35].

### 3.1. The HPA Axis

The HPA and ANS axes have, understandably, received the most scrutiny relative to other endocrine systems that might potentially be affected by ELS [2]. Early-life stress effects on HPA axis functioning may derail normal development during critical periods and potentially persist into adulthood. However, studies have produced mixed results. Numerous studies have shown that ELS is positively associated with HPA axis hyperactivity in both healthy adults and individuals with depression and PTSD, as indicated by increased peripheral cortisol and dehydroepiandrosterone (DHEA) levels, enhanced cortisol awakening response (CAR), or increased ACTH and cortisol responses to environmental stressors or endocrine challenges [36,37,38,39,40,41]. On the other hand, several studies in similar populations and study designs have reported lower peripheral cortisol levels and diminished cortisol responses to psychosocial stressors, which are indicative of HPA axis hypo-activity [42,43,44]. Other studies found no differences as a consequence of ELS exposure in cortisol circadian rhythms [45] or in the CAR [46], and no correlation between ELS and HPA axis reactivity to either a psychological or physiological acute stressor [47]. In a recent study, a tendency towards lower levels of hair cortisol concentration was shown in individuals who reported physical neglect during their childhood [48]. Finally, the results of a recent meta-analysis regarding the relation between ELS and cortisol were enlightening. Specifically, no significant relationships were found between ELS and the CAR, the baseline cortisol, the non-stressed cortisol over time, and the cortisol reactivity [49]. However, in those who had experienced ELS that involved sexual, physical, or emotional abuse, the CAR was heightened, and also, if blood samples were collected, ELS was associated with a blunting effect of cortisol [49]. Therefore, several factors may yield different biological findings, such as (1) distinct definitions of ELS (e.g., objective versus subjective indicators); (2) the nature of ELS (sexual versus physical versus emotional abuse versus neglect), number of episodes, cumulative period of the adverse events, age at first incidence of abuse/neglect, and chronicity; (3) the presence of psychosocial support; (4) the presence of later traumatic events; (5) genetic and epigenetic factors; (6) the sex of the individual; and (7) the outcomes assessed (e.g., phasic, such as diurnal salivary cortisol and cortisol reactivity to challenge vs. tonic cortisol values such as hair cortisol) [45,50,51,52]. 

However, the exact timing of ELS is probably the most important factor modulating HPA axis activity later in life [2]. More specifically, infancy and early childhood (0–5 years of age) are one the most vulnerable periods of brain development [53]. The HPA axis, after an initial hyper-responsive period, may later transition into a stress hypo-responsive period, indicated by decreased basal and stress-induced cortisol levels [51,54]. Several longitudinal studies have shown that throughout early childhood, stress responsivity may decrease with age [53,55,56]. The aforementioned shift from a hyper- to a hypo-responsive HPA axis during the first 5 years of life represents a crucial period regarding the HPA axis programming. Precisely, ELS exposure within the first 2 years in life has been associated with prolonged cortisol reactivity to an acute social stressor among adolescents [57,58]. Thus, ELS exposure together with elevated glucocorticoids levels during the hypo-responsive period could lead to glucocorticoid receptor insensitivity over time, thus affecting the development of a hypo-responsive HPA axis [53]. Moreover, a later particularly sensitive developmental period is adolescence. During this developmental stage, the HPA axis transitions from a period of decreased activity into a hyper-responsivity phase characterized by progressive higher basal and reactive cortisol levels [55,59,60,61]. The effect of ELS on HPA axis basal activity and reactivity during adolescence has been suggested to be opposite than in infancy, as indicated by lower baseline cortisol and blunted cortisol responses to psychosocial stressors [58,62]. It is supposed that these age-related differences in the impact of ELS on HPA axis activity and reactivity play a role in the risk of developing a specific mental disorder later in life. For example, trauma exposure in early childhood is associated with equal risk of developing major depressive disorder or PTSD later in adulthood, whereas if traumatization occurs during adolescence, the risk of developing PTSD has been shown to be greater than that of developing depression [7].

### 3.2. Locus Ceruleus/Autonomic Nervous System (LC/ANS) 

The activity of the LC/ANS and the HPA axis is normally complementary. The regulation of the HPA axis depends partly on vagal influences from the LC/ANS [34]. The majority of studies assessing autonomic activity in adults after early life traumatic experiences, including individuals with PTSD, repeatedly suggest an increased sympathetic and/or decreased vagal activity after a traumatic experience [34]. For example, higher catecholamine responses to psychological stress in police recruits [63], higher rates of syncope frequency in adults exposed to ELS [64], and blunted cardiac output reactivity and increased vascular resistance in response to stress in adolescents with ELS experience [65] have been reported. Moreover, regarding pediatric populations, significantly higher diurnal urinary concentrations of catecholamines in sexually abused girls [66] and continuously higher catecholamine levels 6 months after trauma exposure (motor vehicle accident) have been reported [67]. Elevated catecholamine secretion has been implicated in excessive consolidation of traumatic memories, which is substantial for the development and maintenance of re-experiencing symptoms of PTSD such as intrusive memories, flashbacks, and repetitive nightmares [68,69]. Importantly, the administration of pharmacological agents such as β-blockers immediately after trauma exposure, might be helpful as secondary preventive agents for the development of specific PTSD symptoms [69,70].

### 3.3. Imaging Findings

There is a large body of studies assessing structural and functional brain correlations to ELS. Early-life stress has been associated with reduced volume of corpus callosum, insula, dorsolateral prefrontal cortex (PFC), orbitofrontal cortex (OFC), anterior cingulate gyrus, and caudate, as well as decreased cortical thickness of medial and lateral prefrontal and temporal lobe regions and reduced overall brain volume in humans [71,72]. There have also been numerous studies reporting an association between ELS or childhood trauma and reduced hippocampal volume in adulthood [73]. A recent meta-analysis of structural MRI studies reported reduced grey matter in the right dorsolateral prefrontal cortex and right hippocampus amongst adults with a history of childhood trauma [74]. Nevertheless, the volume of the hippocampus seems to be unaffected in children with childhood-maltreatment-related PTSD [75]. The unaffected hippocampal volume in children but not in adults with maltreatment-related PTSD suggests an initially volumetrically normal hippocampus with subsequent abnormal disrupted development [75]. Regarding the amygdala, functional MRI studies have shown that ELS and childhood trauma are associated with amygdala hyper-responsiveness, particularly to negative-emotion-related stimuli [73,76,77]. It has also been suggested that amygdala hyperactivity as a result of ELS or childhood trauma may mediate the risk for depression later in adult life [78,79]. However, regarding the effect of ELS or childhood trauma on amygdala volume, some studies have reported reduced volume [73] and others greater amygdala volume (in macaque) [80], and some have found effects that depend on the specific type of ELS [81].

Taken together, the aforementioned findings give prominence to the programming effects of ELS on neural networks and endocrine systems involved in stress, especially during vulnerable periods of development. The neuropsychobiological changes following ELS exposure are suggested to mediate risk for adult maladjustment and psychopathology, including PTSD. Understanding the pathways susceptible to alterations as sequelae of ELS could provide new insights into the pathophysiologic trajectories and the longitudinal course of PTSD, with possible implications for therapeutic interventions for stress-related disorders.

## 4. HPA Axis and ANS Alterations in Pediatric PTSD

Chronic stressors such as child maltreatment (i.e., physical and/or sexual abuse or neglect) and acute stressors, such as accidents and earthquakes, are qualitative different and potentially produce qualitatively and quantitatively distinct clinical symptoms and neuroendocrine alterations [82]. Child maltreatment often co-exists with parental psychopathology, which may represent an additional innate biological risk factor, and several psychosocial risk factors, such as low socioeconomic status, unemployment, parental stress, and drug or alcohol abuse [83]. Moreover, it has been suggested that greater dissociation may be related to repeated rather than single trauma [84]. Interestingly, relatively weak independent associations have been shown between a single childhood trauma type (e.g., emotional abuse, physical abuse, sexual abuse, emotional neglect, physical neglect) and health-related quality of life, depression, and PTSD in adulthood [83]. However, there is a cumulative adverse effect of different types of childhood trauma on adult PTSD and depression symptoms with increasing levels of exposure [83]. 

Numerous studies have investigated the neuroendocrine alterations in adults with PTSD. The majority of them have demonstrated increased basal levels of corticotropin-releasing hormone (CRH) in cerebrospinal fluid (CSF) [85,86], decreased cortisol levels at several points during the circadian cycle [87], and reduced salivary/urinary cortisol concentrations [88,89,90]. Nevertheless, several studies have demonstrated divergent findings including increased urinary cortisol excretion [91] or plasma/urinary cortisol concentrations comparable to the non-PTSD individuals [90]. Regarding the SNS, urinary, plasma and CSF catecholamine levels have been consistently found to be increased in adult patients with PTSD [92].

Children and adolescents may exhibit different physiological responses to acute or chronic stressors compared to adults [84]. More specifically, sexually abused girls showed elevated 24 hour catecholamines and their metabolites’ concentrations in urine compared to matched controls [93]. They also exhibited lower evening basal and ovine CRH-stimulated plasma adrenocorticotropic hormone (ACTH) levels, whereas the response of plasma cortisol to CRH injection was comparable to non-abused controls [66]. Moreover, children with a history of maltreatment who attended a day camp research program exhibited significant variation in average morning/afternoon cortisol concentrations depending on the specific type of trauma. In details, morning salivary cortisol concentrations were significantly elevated in children who had undergone physical and sexual abuse and children who had been neglected or emotionally abused [94]. On the other hand, children who had experienced only physical abuse exhibited lower morning cortisol and a smaller decrease in cortisol abundance from morning to afternoon [94]. The aforementioned findings provide support to the notion that neuroendocrine patterns depend on the type of maltreatment [95]. Regarding autonomic dysregulation in maltreated adolescents, an asymmetry between salivary alpha amylase (sAA) concentrations, which are indicative of the SNS activity, and cortisol responses to a social stressor has been demonstrated [96]. The authors concluded that maltreatment status may cause asymmetry between the SNS and the HPA axis by interrupting the synchrony between the two stress system components or by causing attenuation in the one of them but not the other [96]. Moreover, a study in maltreated girls aged between 12 and 16 years old showed that in response to the Trier Social Stress Test (TSST), the maltreated group exhibited an attenuated response of the HPA axis, whereas the control group exhibited an increase in cortisol levels following the TSST and a gradual flattening over time [97]. However, the blunted reactivity of the maltreated youth was not associated with major depressive disorder or PTSD symptoms [97]. Furthermore, a long-term longitudinal study which assessed non-stress cortisol at six time-points, from age 6 to age 30, in girls with confirmed familial maltreatment, demonstrated that the linear trend for abused females was significantly less steep [98]. These findings provided support for the notion that cortisol hyposecretion may follow a period of heightened cortisol secretion (attenuation hypothesis) in victims of abuse [98]. 

There is limited evidence regarding the pathophysiology of childhood PTSD after an acute stressor. In a sample of adolescents who lived at different distances from the epicenter of 1988 earthquake in Armenia, it was demonstrated that the more symptomatic adolescents living in the city closer to the epicenter had lower mean baseline morning cortisol levels, greater Day 2 cortisol suppression following dexamethasone, and a more rapid decline in 3-methoxy-4-hydroxyphenylglycol (MHPG) levels over the course of Day 1 [99]. Interestingly, in terms of PTSD symptoms, only severity of intrusion symptoms was correlated with basal morning cortisol levels and percent suppression by dexamethasone, which is indicative of the fact that in chronic PTSD, repeated distress episodes due to persistent intrusion may over time alter HPA axis function [99]. The opposite may also be true, since there is evidence that acute administration of stress-level doses of glucocorticoids can impair declarative memory retrieval processes [69,100]. On the other hand, it has been shown that acute glucocorticoid administration may reduce recall of trauma-related memory in adult PTSD, even in patients who have chronically elevated glucocorticoid levels [68,101]. 

In a study conducted by our research group, children and adolescents aged 7–18 who had been involved in motor vehicle accidents (MVAs) were assessed immediately after hospitalization, and 1 and 6 months later [67]. Those who developed PTSD had higher plasma noradrenaline concentrations at Months 1 and 6, and higher evening salivary cortisol concentrations at Month 1 compared to the non-PTSD and the control groups [67]. The subjects with a PTSD diagnosis at both Months 1 and 6 also had significantly elevated plasma noradrenaline levels at Month 6 compared to those of Month 1, whereas the initially elevated salivary cortisol concentrations normalized at Month 6 [67]. Moreover, in a study of children (5–17 years of age) who had sustained sexual abuse there was no significant difference between individuals with or without PTSD regarding ACTH and serum cortisol levels [102,103]. However, cortisol levels decreased with increasing time after trauma in the PTSD group, supposedly in the context of an adaptive process preventing adverse effects of prolonged glucocorticoids exposure [102,103]. Finally, a study in female adolescents with single-sexual-trauma-related PTSD found lower dehydroepiandrosterone sulphate (DHEA-S) levels and higher cortisol/DHEA-S ratios in the patient group compared to healthy controls [104]. No significant differences regarding cortisol levels were reported, and there was no correlation between PTSD symptoms and cortisol/ DHEA-S, DHEA-S, or cortisol levels [104]. 

These findings are evidence for HPA axis alterations and SNS dysregulation in pediatric PTSD. These alterations, which are suggested to play a prominent role in the pathophysiology of pediatric PTSD, may represent a neurobiological risk factor leading to further psychopathology in adult life [84]. The implications of these findings regarding the neurobiological natural history of pediatric PTSD are discussed in the following section. 

## 5. The Longitudinal Course of Pediatric PTSD after Accidents 

A study of children exposed to a variety of traumatic injuries demonstrated that elevated levels of urinary catecholamines and cortisol immediately following trauma exposure were positively related to acute and 6 week post-trauma PTSD symptoms [105]. Contrarily, in a study of adult motor vehicle accident victims, the initial urinary cortisol and norepinephrine levels were found to be lower in those who later developed PTSD [106]. These differential hormonal alterations in children compared to adults following trauma exposure were replicated in a study of children after a variety of traumatic events (i.e., motor vehicle accidents, falls, sports related injuries, burns) [107]. In this study, elevated urinary cortisol levels during hospitalization, immediately after trauma, were significantly related to subsequent 6 week PTSD symptoms in children, although at 7 months post-trauma, this relationship remained significant only in boys [107]. Similarly, in a sample of children (8–18 years of age) who had undergone traumatic injuries, including motor vehicle/bicycle accidents, significant correlations between urinary cortisol levels at hospital admission, emotional numbing, and re-experiencing were demonstrated 6 weeks post-trauma, but not 6 months later [108]. 

The longitudinal course and interactions between the HPA axis and the arousal/sympathetic system were described in a study of children and adolescents involved in motor vehicle accidents [67,109]. In this study, 30% of the participants had developed PTSD 1 month after the accident, whereas, 15% of the initial population maintained their PTSD diagnoses 6 months later. The group that developed PTSD 1 month after the accident and maintained their PTSD diagnoses 6 months later (the longitudinal PTSD group) was compared with a group that did not develop PTSD after being exposed to an accident (non-PTSD). A group of non-traumatized children served as the control group. Daily salivary cortisol profiles (at five time-points), morning serum cortisol, interkeukin-6 (IL-6), and plasma catecholamines (norepinephrine (NE), epinephrine (E) and dopamine (D)) were measured at three time-points: immediately after the accident, and 1 and 6 months later. Evening (9pm) salivary cortisol and morning (8:00 am) interleukin-6 (IL-6) levels were both predictive of PTSD development at Month 6 [109]. Plasma NE concentrations did not differ among groups after the accident, and they were not predictive of later PTSD development [109]. The PTSD group exhibited higher NE concentrations compared to the non-PTSD and the control groups at Months 1 and 6 [67]. More importantly, NE levels became gradually higher (from Month 1 to Month 6) within the PTSD group [67]. Similarly, within the non-PTSD group, NE was increased from Month 1 to Month 6, at a lower setting, showing that sub-threshold symptoms of PTSD may also be related to milder NE elevations [67]. In the longitudinal PTSD group, as circadian rhythm and evening cortisol normalized 1 month after the accident, NE elevations became greater, which could have been the effect of lifting a cortisol-mediated noradrenergic system restraint [67]. This study suggested that in young individuals exposed to trauma, an initial increase in cortisol concentrations may be followed by a state of low cortisol, as time passes from the traumatic event. At the same time, interacting with the HPA axis, NE concentrations become progressively elevated. This longitudinal interaction between the HPA and the SNS axes seems to characterize those who develop and maintain PTSD diagnosis over time. 

Clinical symptoms of PTSD may thus be attributed to cortisol decrease that fails to shut down the catecholarminergic response in limbic structures. It has been suggested that an exaggerated catecholamine secretion is related to excessive consolidation of traumatic memories, which in turn may lead to the development and maintenance of PTSD symptoms, such as intrusive thoughts, flashbacks, and repetitive nightmares [67,68,70]. By contrast, glucocorticoids impair or have little effect on memory consolidation in the absence of arousal-induced noradrenergic activation [69,100].

The pattern of heightened baseline catecholamines and enhanced HPA axis feedback, which can sometimes result in lower cortisol levels in adults with PTSD than in non-PTSD individuals, may be the result of a time-dependent stress system dysregulation beginning from childhood [67,109]. Although HPA axis dysregulation has been recognized as a common pattern among adults as well as children diagnosed with PTSD, a definite conclusion regarding the longitudinal course of pediatric PTSD cannot be determined, given the divergence of the findings regarding stress hormone secretion. More specifically, low cortisol concentration is not a persistent finding among adults with PTSD, since several investigations have not demonstrated significant findings or presented circumstantial associations between PTSD and HPA axis dysregulation [110]. Moreover, altered HPA axis functionality observed in PTSD across lifespan may represent a pre-trauma risk factor for the disorder, indicating the presence of a vulnerable predisposition due to genetic and environmental risk factors, such as exposure to ELS during development, occurring prior to a traumatic event [110]. On the other hand, some researchers suggest that the alterations in physiological mechanisms found in PTSD may be the result of the disorder itself, or may even be attributed to trauma exposure rather than PTSD per se [110,111]. 

## 6. The Role of Epigenetics

Epigenetic modifications are changes, resulting from environmental factors and to some extent reversible, that can regulate functional gene expression in a potentially inter-generationally transmissible manner [112]. A number of studies have indicated that adverse early experiences may contribute to the installment of epigenetic marks that affect stress responses [113]. Moreover, there is an increasing body of evidence for the role of specific genes’ epigenetic alterations in the pathophysiology of PTSD [50,114]. Recently, it as shown that gene expression profiles of PTSD patients with matched adult trauma experience but different childhood adverse events are 98% non-overlapping [115]. Furthermore, these gene expression changes were mediated by DNA methylation changes to a much greater degree in the childhood abuse group [115]. These results suggest that epigenetic mechanisms, which exert greater impact during early development, may mediate differences in the pathophysiology of adult PTSD, depending on preceding exposure to childhood maltreatment [115]. In particular, some glucocorticoid-related genes such as the *NR3C1* and the glucocorticoid receptor gene promoter 1F, are subject to stress-related epigenetic regulation throughout life [116,117]. The effects of in utero stress on the infant neurobiology, the association of maternal PTSD with the increased risk of PTSD development in the offspring, and the relevance of ELS to the development of PTSD may also be compatible with epigenetic explanations [118]. More precisely, it has been shown that stress in utero may contribute to PTSD vulnerability mainly through elevations of glucocorticoid levels, influences on fetal brain development, and programming of the HPA axis [34]. One study of mothers exposed to the World Trade Center (11 September 2001) attacks during pregnancy and their 1 year old babies demonstrated an association between maternal PTSD and both awakening and bedtime cortisol levels in their babies’ saliva [119]. Furthermore, babies born to mothers with PTSD had lower cortisol values compared to the non-PTSD group [119]. 

**Figure 1:**
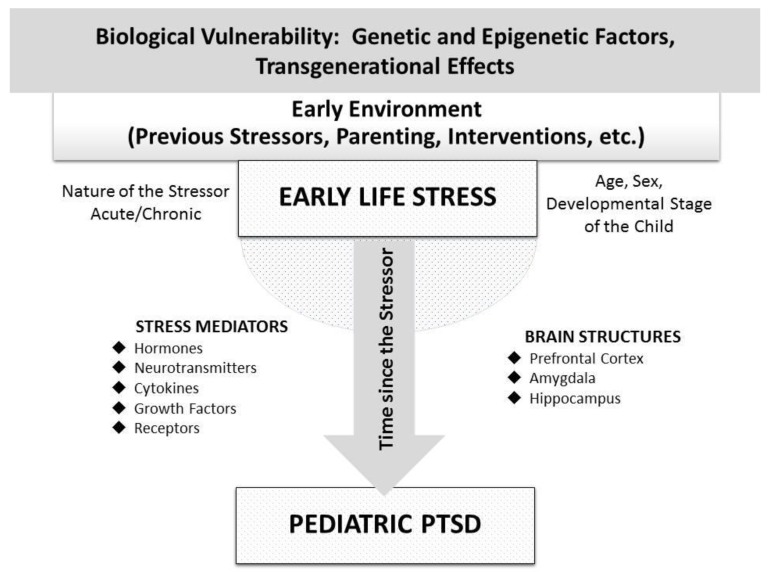
Parameters affecting the neurobiological trajectories from early-life stress to pediatric PTSD.

In conclusion, epigenetic alterations provoked by ELS exposure may play a central role in the long-term and transgenerational biological trajectories of pediatric PTSD, mainly through the disruption of developmental programming of stress-related structural and molecular neurobiological pathways. Figure 1 depicts the parameters contributing to diverse neuroendocrine findings in pediatric PTSD following ELS, as well as the main neurobiological mediators connecting ELS with pediatric PTSD.

## 7. Conclusions

A large body of evidence underscores the crucial role of the stress system and its mediators in the pathophysiology of PTSD in children and adults. These chronic neurobiological alterations (e.g., chronic hypo-cortisolism and high catecholamines) are associated with metabolic, behavioral, and cardiovascular implications that may be related to increased morbidity and mortality in individuals with PTSD. Finally, ELS exposure during vulnerable periods of neurodevelopment is one of the most important factors modulating stress system activity and plays a crucial role in the risk of developing a mental disorder later in adult life. Thus, early interventions are essential for the promotion of mental and physical health of individuals suffering from conditions and disorders related to chronic stress.
